# First report of *Ancylostoma tubaeforme* in Persian Leopard (*Panthera pardus saxicolor*)

**Published:** 2010-03

**Authors:** MR Youssefi, SH Hoseini, SM Hoseini, BA Zaheri, M Abouhosseini Tabari

**Affiliations:** 1 Dept. of Veterinary Parasitology, Islamic Azad University Babol–Branch, Iran; 2 Dept. of Veterinary Parasitology, Faculty of Veterinary Medicine, Tehran University of Medical Sciences, Iran; 3 Centre of Environment Biology, Semnan, Iran; 4 Dept. of Veterinary Pharmacology, Faculty of Veterinary Medicine, University of Tehran, Tehran, Iran

**Keywords:** *Ancylostoma tubaeforme*, Hookworm, Leopard, Iran

## Abstract

*Ancylostoma tubaeforme* was originally described as a separate species parasitizing the cat. The adults of *A. tubaeforme* are 7 to 12 mm long. *A. tubaeforme* can be differentiated from the adults of *A. braziliense* and *A. ceylanicum* by the presence of three teeth. Here we describe the first report of *A. tubaeforme* in a Persian young female leopard, 2–3 years old, with head and trunk length 120 centimeters, length of tail 98 centimeters and body weight 35 kilograms.

## Introduction

*Ancylostoma tubaeforme* as a separate species parasitizing the cat was originally described by Zeder in 1800(1). It was finally given a firm position as a separate species within the genus by Burrows (1962), who compared the adults of *A. ubaeforme* with those of *A. caninum.* This worm is found throughout the world, wherever there are domestic cats (2).

The adults of *A. tubaeforme* are 7 to 12 mm long. To distinguish the adult specimens of hookworms in cat, the differentiation should be based on the buccal capsule shape. First, members of the genus *Ancylostoma* can be separated from those of *Uncinaria* by determining whether there are ventral teeth in the buccal capsule. Specimens of *Ancylostoma* have large teeth within the buccal capsule, while specimens of *Uncinaria* are recognized by the presence of cutting plates.

The adults of *A. tubaeforme* can be differentiated from the adults of *A. braziliense* and *A. ceylanicum* by the presence of three teeth on either side of the ventral midline (*A. brazileinse* and *A. ceylanicum* each possess two such teeth) (3, 4). The adults of *A. braziliense* are 4 to 10.5 mm long. The adults of *A. braziliense* and *A. ceylanicum* possess only two teeth on the ventral aspect of the buccal cavity, with the lateral tooth being large and the median tooth quite small. The adults of *A. tubaeforme* have three teeth on each side of the buccal capsule. *A. braziliense* can be differentiated from *A. ceylanicum* by careful examination of the teeth within the buccal cavity. The medial teeth are smaller in *A. braziliense* than they are in *A. ceylanicum* (1). Another means of separating these two species is by careful examination of the copulatory bursa of the male. The lateral lobes of the bursa are relatively shorter in *A. ceylanicum* than they are in *A. braziliense,* and the branching of the externo-dorsal rays occurs more posteriad in *A. ceylanicum* than it does in *A. brazileinse* (5).

In the present study, we describe the detection of *A. tubaeforme* in a leopard (*Panthera pardus saxicolor*) in Iran, which is the first report of such infected leopard in Iran.

## Case Report

The Persian leopard is said to be one of the largest of all the subspecies of leopards in the world. The leopard is the smallest of the great cats (lion, tiger, and jaguar). Males are up to 50% larger than the females. A young female leopard 2–3 years was shot accidentally by villagers in Ahovan County around of Damghan City (Latitude 36.083, longitude 58.967 and elevation1238 meters, East Semnan province). Three days after death, its carcass was frozen and transferred to the Department of Veterinary Parasitology of the Tehran University, Iran. We examined the digestive tract for endoparasits by sceening (Mesh 70). The specimens were fixed and preserved in 70 % ethanol. They were cleared in lacto phenol and studied in temporary mounts. Confirming the identification, samples were sent for researcher of Veterinary Parasitology museum, Tehran University. Five nematode helminthes, which were obtained, identified as *A. tubaeforme*. All samples were female and measured about 6.7±0.2 millimeters. The eggs of *A. tubaeforme* have been measured to be 55–76 by 34–45 µm with means of 61 by 40 µm.

**Fig. 1:**
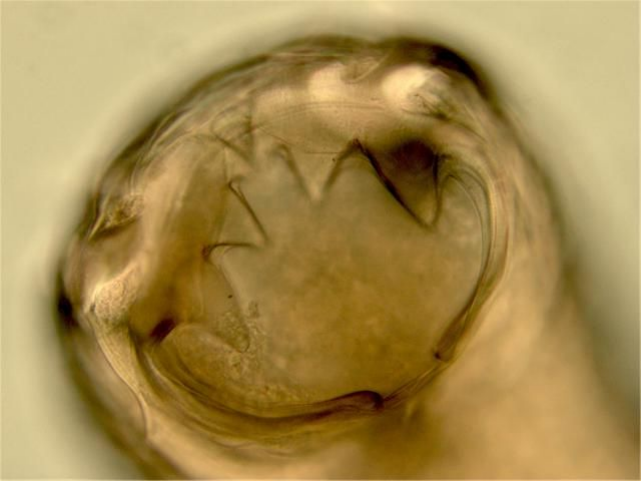
Head of *Ancylostoma tubaeforme* derived from (*Panthera pardus saxicolor*) in Iran
